# The Pokeweed Leaf mRNA Transcriptome and Its Regulation by Jasmonic Acid

**DOI:** 10.3389/fpls.2016.00283

**Published:** 2016-03-16

**Authors:** Kira C. M. Neller, Alexander Klenov, Katalin A. Hudak

**Affiliations:** Department of Biology, York University, TorontoON, Canada

**Keywords:** *Phytolacca americana*, pokeweed, RNA-seq, transcriptome, jasmonic acid, ribosome inactivating protein, pokeweed antiviral protein, natural antisense transcript

## Abstract

The American pokeweed plant, *Phytolacca americana*, is recognized for synthesizing pokeweed antiviral protein (PAP), a ribosome inactivating protein (RIP) that inhibits the replication of several plant and animal viruses. The plant is also a heavy metal accumulator with applications in soil remediation. However, little is known about pokeweed stress responses, as large-scale sequencing projects have not been performed for this species. Here, we sequenced the mRNA transcriptome of pokeweed in the presence and absence of jasmonic acid (JA), a hormone mediating plant defense. Trinity-based *de novo* assembly of mRNA from leaf tissue and BLASTx homology searches against public sequence databases resulted in the annotation of 59 096 transcripts. Differential expression analysis identified JA-responsive genes that may be involved in defense against pathogen infection and herbivory. We confirmed the existence of several PAP isoforms and cloned a potentially novel isoform of PAP. Expression analysis indicated that PAP isoforms are differentially responsive to JA, perhaps indicating specialized roles within the plant. Finally, we identified 52 305 natural antisense transcript pairs, four of which comprised PAP isoforms, suggesting a novel form of RIP gene regulation. This transcriptome-wide study of a Phytolaccaceae family member provides a source of new genes that may be involved in stress tolerance in this plant. The sequences generated in our study have been deposited in the SRA database under project # SRP069141.

## Introduction

The pokeweed plant, *Phytolacca americana*, is a member of the Phytolaccaceae family of flowering plants that includes 65 species of herbs, shrubs, and trees. Pokeweed is native to eastern North America and has become naturalized in Europe, the West Indies and Asia. This species is of interest because it synthesizes PAP, a RIP with RNA *N*-glycosidase activity. Several isoforms of PAP are reported to exist in pokeweed, exhibiting different temporal (PAP-I, PAP-II, PAP-III) and spatial (PAP-S, PAP-R, PAP-alpha) expression patterns (Irvin, 1975; Irvin et al., 1980; Barbieri et al., 1982; Bolognesi et al., 1990; Kataoka et al., 1992; Rajamohan et al., 1999). RIPs are present in less than 20% of angiosperm taxonomic orders and phylogenetic analysis indicates a complex evolutionary history (Di Maro et al., 2014). They are potent defense proteins effective against a range of viruses, fungi, and less commonly, insects (Stirpe, 2013).

Pokeweed has broad applications in agriculture and medicine. PAP inhibits the replication of several plant and animal viruses, either through ribosome inactivation which limits viral proliferation or by direct depurination of the viral genome (Lodge et al., 1993; Rajamohan et al., 1999; He et al., 2008; Karran and Hudak, 2008; Mansouri et al., 2009). Interestingly, recent work demonstrates that pokeweed accumulates high levels of heavy metals, especially cadmium and manganese, with promising applications in soil detoxification (Dou et al., 2009; Zhao et al., 2011, 2012). Nevertheless, little is known about pokeweed, as the genes involved in stress response have not been identified.

Here, we have sequenced the pokeweed mRNA transcriptome in the presence and absence of JA treatment. JA is a plant hormone that mediates defense against herbivores and necrotrophic pathogens. As herbivores are often viral vectors, the JA pathway also has important implications for virus resistance. We showed recently that PAP mRNA and protein levels increase in the presence of JA (Klenov et al., 2015). A link between jasmonate and other plant RIPs has previously been established. For example, PIP2 from *Phytolacca insularis*, ME1 from *Mirabilis expansa* and JIP60 from barley are induced by JA or its methyl jasmonate derivative (Dunaeva et al., 1999; Song et al., 2000; Vepachedu et al., 2003). Furthermore, expression of the insecticidal maize RIP2 is increased 100-fold at the RNA level upon caterpillar feeding, demonstrating the relevance of RIPs in anti-herbivory (Chuang et al., 2014). By sequencing the transcriptome of pokeweed treated with JA, we will gain novel information about the regulation of specific PAP isoforms and how these proteins are integrated within the larger network of pokeweed pathogen response. This work lays the important foundation to understand the resiliency of pokeweed to biotic and abiotic factors.

We report the *de novo* assembly and annotation of the pokeweed mRNA transcriptome from leaf tissue. Through a combination of differential expression and GO analysis, we identified JA-responsive genes and enriched GO terms involved in stress and defense. We confirmed the existence of several published PAP isoforms, reported their distinct responses to JA and cloned a potentially novel PAP isoform. Finally, we report the discovery of PAP NATs that are also JA-responsive, which may represent a novel form of RIP gene regulation.

## Materials and Methods

### Pokeweed Growth Conditions and Jasmonic Acid Treatment

Pokeweed seeds were treated with 37% sulfuric acid for 5 min and submerged in water for 4 days at room temperature. Seeds were germinated in soil (Promix BX) and maintained in a growth chamber (AC60, Biochambers, Winnipeg, MB, Canada) under fluorescent and incandescent lights at 180 μE m^-2^ s^-1^ with periods of 16 h day and 8 h night. Fertilizer was provided once every 2 weeks with N:P:K 20:20:20. For experimental treatment, plants were sprayed with 5 mL of 5 mM JA dissolved in 0.5% ethanol (to improve the solubility of JA). Negative control plants were sprayed with 0.5% ethanol. Following treatment, plants were returned to the chamber and leaf tissue was harvested 24 h later. All plants used in this study were at the 4-leaf stage of growth.

### Total RNA Isolation, Library Construction, and Sequencing

Total RNA was extracted from leaf tissue of pokeweed plants treated with 5 mM JA in 0.5% ethanol or 0.5% ethanol alone using the RNeasy Plant Mini Kit (Qiagen, Valencia, CA, USA). An equal amount of total RNA from three independent plants was pooled for each biological replicate. In total, six mRNA libraries were generated (*n* = 3 per treatment, from 18 total plants). Libraries were constructed with the TruSeq Stranded mRNA Library Preparation Kit (RS-122-2101, Illumina). Sequencing was performed on a single lane of an Illumina HiSeq 2500 machine by The Centre for Applied Genomics (The Hospital for Sick Children, Toronto, ON, Canada) to generate paired-end reads of 150 bases. Raw sequences are available at the SRA database under project # SRP069141.

### RNA-Seq Data Processing and *De Novo* Transcriptome Assembly

Prior to assembly, adapters were clipped, low-quality bases were trimmed (*Q* < 30, averaged over four bases) and synchronicity of paired-end files was maintained using Trimmomatic v. 0.32.1 (Bolger et al., 2014) as follows: PE -phred33 ILLUMINACLIP:TruSeq3-PE.fa:2:30:10 SLIDINGWINDOW:4:30. The pokeweed transcriptome was assembled with Trinity v. r2014-04-13p1 (Grabherr et al., 2011) using the following command-line, which invoked paired-end, stranded information: –seqType fq –JM 48G –left reads-1.fq –right reads-2.fq –SS_lib_type RF –CPU 24.

### Transcriptome Annotation and Refinement

Trinotate v. 2.0.1 (Haas et al., 2013) was used for transcriptome annotation. BLAST (Altschul et al., 1997) searches were conducted against both the SwissProt (Bairoch and Boeckmann, 1991) and UniRef90 (Suzek et al., 2007) databases (current as of January, 2015). Owing to strand-specific sequencing, only the plus strand of the transcriptome was queried with BLASTx. Transdecoder-predicted and translated ORFs were queried with BLASTp. The *E*-value threshold was set to 0.001 and only the top-scoring hit was retained. HMMER v. 3.1b2 (Finn et al., 2011) was used to search for conserved protein domains in predicted ORFs against the pfam-A (Finn et al., 2014) database (current as of January, 2015). BLAST homologies and Pfam domain entries were loaded into the pre-formatted Trinotate SQLite database which contained UniProt-associated annotation information.

The complete, Trinity-assembled transcriptome (*Raw* assembly) was filtered to retain only transcripts expressed at an abundance of 1 FPKM (*Filtered* assembly) or only BLASTx-annotated transcripts (*BLASTx* assembly). Assembly statistics were calculated and transcript coverage of each top-scoring unique hit was determined with custom scripts that came bundled with Trinity software. A local installation of the Galaxy platform (Giardine et al., 2005; Blankenberg et al., 2010; Goecks et al., 2010) was used for manipulation of large datasets.

### Transcript Abundance and Differential Expression Analysis

Transcript-level rather than unigene-level expression was investigated in order to retain isoform-specific information. Reads from individual libraries were aligned to the reference transcriptome with bowtie v. 1.1.1. (Langmead et al., 2009) and quantified by RSEM v. 1.2.18 (Li and Dewey, 2011). A table of TMM-normalized FPKM expression values and a separate table of raw fragment counts were generated with custom scripts.

Differentially expressed transcripts were identified from raw counts with the Bioconductor package EdgeR v. 3.1 (Robinson et al., 2010) in the statistical program R (R Development Core Team, 2011). Three biological replicates for each condition were provided. A subset of differentially expressed transcripts (FDR < 0.001 and FC ≥ 4) was extracted and used to generate a heatmap of hierarchically clustered, log_2_-transformed and median-centered FPKM values. All scripts came bundled with Trinity software and default parameters were used, supplemented with the strand-specific parameter -SS_lib_type RF when applicable.

### Gene Ontology Analysis

Blast2GO v. 3.0 (Conesa et al., 2005) was used to map GO terms to parent plant GOSlim terms in order to obtain a broad overview of the transcriptome. To identify enriched terms, a Fisher’s test was conducted in Blast2GO with FDR < 0.001. For NAT GO term enrichment, any NAT pair with at least one protein-coding transcript, as annotated by BLASTx, was included in the test set. For enrichment of JA-responsive NAT pairs, in addition to the above protein-coding requirement, only pairs with differentially expressed sense and antisense transcripts were included in the test set. The raw, Trinity-assembled pokeweed transcriptome served as the reference set for all Fisher’s tests conducted in this study.

### Identification of PAP Isoforms and Natural Antisense Transcripts

Following transcriptome annotation, any transcript that matched a published PAP sequence as its top BLASTx hit and contained a predicted RIP protein domain was considered to be a PAP isoform. To identify NATs, a BLASTn search was conducted whereby the plus strand of the complete transcriptome was aligned to a local database containing the reverse complement of all transcripts. The BLASTn *E*-value threshold was set to 0.001.

### Cloning of Novel PAP Isoform and PAP Natural Antisense Transcript

All primer sequences used in this study are available in Supplementary Data Sheet 1. Reverse transcription was performed on 500 ng of total pokeweed RNA in a 20 μL reaction volume containing 5 mM DTT, 1 μM reverse primer, 1X First Strand Buffer (50 mM Tris-HCl pH 8.3, 75 mM KCl, 3 mM MgCl_2_), 0.5 mM dNTPs, 20 units Murine RNase Inhibitor (NEB) and 25 units Superscript III reverse transcriptase (Thermo Fisher). The reaction was incubated at 42°C for 1 h and heat inactivated at 70°C for 20 min.

Following cDNA synthesis, a PCR reaction was conducted, containing 1X Q5 buffer (NEB), 0.5 μM forward primer, 0.5 μM reverse primer, 200 mM dNTPs, 2 μL cDNA and 1 unit Q5 DNA polymerase (NEB) in a total volume of 50 μL. The PCR program included an initial denaturation of 95°C for 30 s, 30 cycles of 95°C for 30 s, 58°C for 30 s, 72°C for 120 s and finished with an extension at 72°C for 180 s. PCR products were separated on low-melt agarose and the correct size band excised and purified with EZ-10 Spin columns (Biobasic). The purified product was digested with BamHI and SalI, ligated into pBS-KSII and sequenced.

### qRT-PCR Validations

For qRT-PCR, the reverse transcription step was performed in the same manner as cloning except that 2 μg of total pokeweed RNA from either control or JA treated plants was used with reverse primers corresponding to a specific transcript or 28S rRNA as the internal control. The qPCR reaction contained 5 μL of cDNA, 0.7 μM forward primer, 0.7 μM reverse primer and 1X SYBR Green Mastermix (Clontech). Each reaction was split into three technical replicates and analyzed in a Qiagen Rotor-gene-Q real time PCR cycler. *C*t-values were calculated with the ΔΔ*C*t relative quantification method. Three biological replicates were conducted for each transcript. For statistical analysis, a one-tailed, unpaired Student’s *t*-test was conducted using GraphPad Prism v.5.01.

## Results

### Assembly and Annotation of the Pokeweed mRNA Transcriptome

An overview of the entire study is provided in Supplementary Figure S1. A total of 406,995,054 high-quality reads from control and JA-treated pokeweed plants were combined and the mRNA transcriptome was assembled with Trinity software. Assembly statistics are provided in **Table 1**. The complete pokeweed transcriptome (*Raw*) contained 216,891 transcripts belonging to 177,709 unigenes. To identify contigs expressed at a reasonable threshold, the *Raw* assembly was filtered to retain only those having a minimum abundance of 1 FPKM; this reduced the number of transcripts and unigenes to 89,682 and 77,731, respectively (*Filtered*). The *Raw* assembly was also filtered on the basis of BLASTx annotation, which resulted in 59,096 and 38,291 annotated transcripts and unigenes, respectively (*BLASTx*). Furthermore, 16,245 unique proteins were represented by transcripts with at least 70% BLASTx alignment coverage, indicating a high-quality transcriptome assembly (Supplementary Data Sheet 2). The Trinotate report for BLASTx-annotated transcripts is available in Supplementary Data Sheet 3. Interestingly, only 38% of transcripts in the *Filtered* assembly were also BLASTx annotated, suggesting that the majority of pokeweed-expressed transcripts are not shared with those from available plants.

**Table 1:** Assembly statistics for pokeweed mRNA transcriptomes.

Parameter	Raw	Filtered	BLASTx
# of Transcripts	216,891	89,682	59,096
# of Genes	177,709	77,731	38,291
Transcriptome size (Mb)	157	74	85
N50 (bp)	1,168	1,617	2,102
Average length (bp)	724	821	1,439
Median length (bp)	406	373	1,159
Minimum length (bp)	201	201	201
Maximum length (bp)	15,776	15,776	15,776
# of Transcripts ≥ 1 Kb	42,541	24,436	32,746

*Raw: the complete, Trinity-assembled transcriptome; *Filtered*: only transcripts ≥ 1 FPKM were retained; *BLASTx:* only BLASTx-annotated transcripts from the UniRef90 and SwissProt databases were retained.*

**FIGURE 1:**
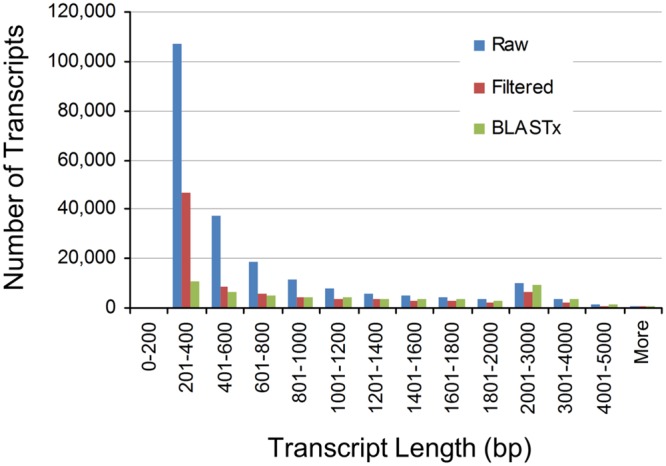
**Length distributions of pokeweed mRNA transcriptomes**.

To assess the contiguity of our different assemblies, we also determined their N50 values (**Table 1**). The N50 statistic is a weighted median such that 50% of the assembly is contained in contigs equal to or larger than this value. N50 values for the *Raw, Filtered*, and *BLASTx* assemblies were 1,168, 1,617, and 2,102 bp, respectively. These values indicated that expressed and annotated transcripts tended to be longer in length, which was confirmed from the assembly length distributions (**Figure 1**). Notably, the *Raw* assembly had a majority of transcripts between 200 and 600 bp in length, while this size class accounted for a smaller proportion of the *Filtered* and *BLASTx* assemblies. These short transcripts could represent partial transcripts, microRNA precursors and/or assembly artifacts.

Functional annotation of the complete, assembled pokeweed transcriptome was carried out with the Trinotate pipeline and GO analysis was conducted using Blast2GO software. GO terms were mapped to corresponding plant GOSlim terms in order to obtain a summary of the transcriptome (Supplementary Figure S2). GOSlims are cut-down versions of the ontology with reduced detail of lower-level terms; they are useful for providing a general overview of the transcriptome. In total, 36,423 transcripts were annotated with 238,251 GO terms, distributed amongst the categories of Biological Process, Molecular Function, and Cellular Component. Within the Biological Process category, the most abundant terms included response to stress (5,471 transcripts; 15.0% of all transcripts), transport (5,365; 14.7%), and cellular protein modification process (5,200; 14.3%). Nucleotide binding (8,403; 23.1%), DNA binding (4,415; 12.1%), and kinase activity (3,219; 8.8%) comprised the majority of terms in the Molecular Function category. Finally, the Cellular Component distribution indicated that most annotated proteins localized to the plasma membrane (4,974; 13.7%), plastid (3,559; 9.8%), or cytosol (2,887; 7.9%). Taken together, GO analysis indicates that the majority of pokeweed transcripts can be grouped into a small number of broad yet distinct functional categories.

### Identification of Jasmonic Acid-Responsive Genes

Following transcriptome annotation, our next goal was to identify JA-responsive genes through differential expression analysis. Briefly, reads from each library were individually aligned back to the complete reference transcriptome and the abundance of each transcript was determined; differential expression analysis was then conducted based on normalized read counts. The most abundant, BLASTx-annotated transcripts from control and JA-treated plants are summarized in **Table 2** and abundances of all transcripts are available in Supplementary Data Sheet 4. Control plants had high expression of several genes encoding photosynthetic proteins, including RuBisCO and photosystem-associated components. Conversely, JA-treated plants had high abundance of transcripts encoding defense proteins, including two defensin-like proteins, two isoforms of PAP and a proteinase, in addition to constitutive plant metabolic proteins.

**Table 2:** Most abundant, BLASTx-annotated transcripts expressed in pokeweed under control (E) and JA treatments.

Transcript ID	Top BLASTx Hit	Gene Name	log_2_FPKM
**E**			
c58232_g7_i1	RBS1_MESCR	Ribulose bisphosphate carboxylase small chain 1, chloroplastic	14.74
c58494_g1_i1	CB2A_SPIOL	Chlorophyll a–b binding protein, chloroplastic	14.73
c111935_g1_i1	PSBR_SOLTU	Photosystem II 10 kDa polypeptide, chloroplastic	14.00
c58232_g6_i1	RBS2_MESCR	Ribulose bisphosphate carboxylase small chain 2, chloroplastic	13.83
c111786_g1_i1	GL33_ARATH	Germin-like protein subfamily 3 member 3	13.09
c47181_g1_i1	GRP1_DAUCA	Glycine-rich RNA-binding protein	12.64
c16825_g1_i2	CAHC_SPIOL	Carbonic anhydrase, chloroplastic	12.58
c111825_g1_i1	CB23_TOBAC	Chlorophyll a–b binding protein 36, chloroplastic	12.57
c60753_g1_i1	CB12_PETHY	Chlorophyll a–b binding protein, chloroplastic	12.27
c111752_g1_i1	PSAK_ARATH	Photosystem I reaction center subunit psaK, chloroplastic	12.18
**JA**			
c112185_g1_i1	DEF_NELNU	Defensin-like protein	16.54
c51788_g1_i1	DF322_SOLTU	Defensin-like protein P322	15.30
c58494_g1_i1	CB2A_SPIOL	Chlorophyll a–b binding protein, chloroplastic	14.68
c58232_g7_i1	RBS1_MESCR	Ribulose bisphosphate carboxylase small chain 1, chloroplastic	14.37
c3137_g1_i1	RIP1_PHYAM	Antiviral protein I	14.35
c111935_g1_i1	PSBR_SOLTU	Photosystem II 10 kDa polypeptide, chloroplastic	14.01
c58232_g6_i1	RBS2_MESCR	Ribulose bisphosphate carboxylase small chain 2, chloroplastic	13.80
c60978_g1_i1	XCP1_ARATH	Xylem cysteine proteinase 1	13.49
c3192_g1_i1	RIP2_PHYAM	Antiviral protein 2	13.18
c47181_g1_i1	GRP1_DAUCA	Glycine-rich RNA-binding protein	12.70

*Transcripts are listed in order of decreasing abundance.*

**FIGURE 2:**
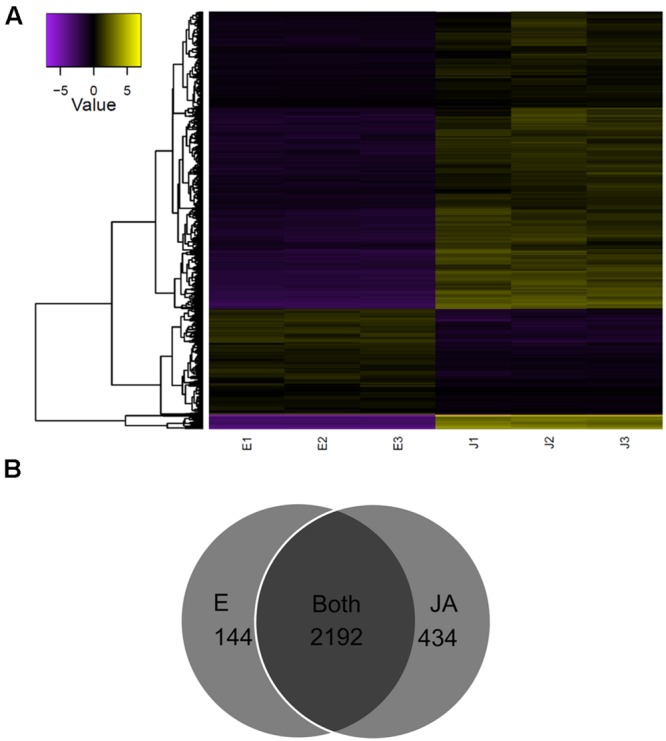
**Identification of JA-responsive genes in pokeweed. (A)** Heat map of expression values (log_2_FPKM, median-centered) of the top differentially expressed transcripts (FDR < 0.001, FC ≥ 4). **(B)** Venn diagram depicting treatment-specific expression patterns of transcripts in **(A)**.

A total of 8,264 transcripts were differentially expressed between control and JA-treated plants (FDR < 0.05; Supplementary Figure S3). A subset of 2,770 (FDR < 0.001, FC ≥ 4) was defined for downstream analysis (**Figure 2A**). Of these, the majority of transcripts increased with JA treatment (2067; 75%). Furthermore, as shown in **Figure 2B**, most of the transcripts were expressed in both control and JA-treated plants (2192; 79%); interestingly, a considerable number was detected only in JA-treated plants (434; 16%). Of these JA treatment-specific transcripts, 165 encoded known proteins as annotated by BLASTx (Supplementary Data sheet 5). Many have well-established roles in defense, including a pathogenesis-related protein, and several chitinases, proteinases, peroxidases, and terpenoid biosynthesis enzymes. JA-induced transcription factor families such as ERF, MYB, and TIFY were identified, as well as two enzymes involved in JA biosynthesis, jasmonate *O*-methyltransferase and 4-coumarate-CoA ligase-like 5. Interestingly, among the most abundant, JA-specific transcripts was a putatively novel isoform of PAP, c115037_g1_i1.

The top JA-responsive, BLASTx-annotated transcripts are summarized in **Table 3** and the complete list of differential expression results is provided in Supplementary Data Sheet 6. As expected, many of these transcripts encoded proteins involved in JA metabolism (Supplementary Figure S4). Additionally, several defense genes were among those most differentially expressed; these included *intracellular ribonuclease LX, nerolidol synthases, antiviral protein alpha*, and *defensin-like protein*. To obtain insight into the functional roles of JA-responsive transcripts in pokeweed, we conducted GO term enrichment analysis (**Table 4**). Up- and down-regulated transcripts were investigated separately in order to determine their independent contributions within the plant. Up-regulated transcripts were highly enriched in terms related to stress and defense responses, indicating marked transcriptional reprogramming in JA-treated plants. Down-regulated transcripts were not enriched in any well-defined stress responses and the enriched terms did not appear to represent any common themes.

**Table 3:** Top JA-responsive, BLASTx-annotated genes in pokeweed.

Transcript ID	Top BLASTx Hit	Gene name	log_2_FC	log_2_FPKM E	log_2_FPKM JA	FDR
c112223_g1_i1	LOX21_SOLTU	Linoleate 13S-lipoxygenase 2-1, chloroplastic	13.97	-4.63	9.58	1.51E–182
c53706_g1_i2	TRPB_CAMAC	Tryptophan synthase beta chain 2, chloroplastic	13.36	-4.18	9.32	5.28E–157
c20579_g1_i1	RNLX_SOLLC	Intracellular ribonuclease LX	13.84	-2.93	11.08	3.64E–148
c112209_g1_i1	NATT3_THANI	Natterin-3	8.67	1.72	10.51	8.82E–144
c3273_g2_i1	NES2_FRAAN	(3S,6E)-nerolidol synthase 2, chloroplastic/mitochondrial	10.63	-2.41	8.34	8.92E–137
c61047_g1_i1	BSPA_POPDE	Bark storage protein A	13.10	-4.08	9.16	2.85E–135
c3273_g1_i1	NES1_FRAAN	(3S,6E)-nerolidol synthase 1	13.30	-6.42	7.29	6.16E–135
c50513_g1_i1	ZOG_PHALU	Zeatin *O*-glucosyltransferase	13.77	-7.38	7.00	3.57E–125
c60944_g1_i1	RIPA_PHYAM	Antiviral protein alpha	12.99	-1.58	11.68	9.32E–120
c112185_g1_i1	DEF_NELNU	Defensin-like protein	13.19	3.30	16.54	3.77E–119

*Transcripts are listed in order of decreasing significance.*

**Table 4:** Top enriched GO terms amongst JA-responsive genes in pokeweed (FDR < 0.001).

Down-regulated transcripts (703)		Up-regulated transcripts (2067)	
GO Term	FDR	GO Term	FDR
Extracellular region	5.96E–04	Response to wounding	1.28E–26
Hydrolase activity, hydrolyzing *O*-glycosyl compounds	2.88E–03	Response to endogenous stimulus	1.82E–16
Heme binding	2.88E–03	Response to jasmonic acid	5.82E–16
Methylammonium transmembrane transporter activity	2.88E–03	Response to chitin	3.90E–15
Cell wall	3.91E–03	Defense response	1.54E–14
Storage vacuole	3.91E–03	Regulation of systemic acquired resistance	2.60E–10
Nitrate reductase (NADH) activity	1.31E–02	Cytoplasm	2.60E–10
Nucleus	1.73E–02	Cellular component organization or biogenesis	6.27E–10
Molybdopterin cofactor binding	1.98E–02	Heme binding	7.75E–10
Oxidation–reduction process	2.02E–02	Nutrient reservoir activity	1.19E–09

*The number in brackets indicates the number of down- or up-regulated transcripts within the test set.*

**FIGURE 3:**
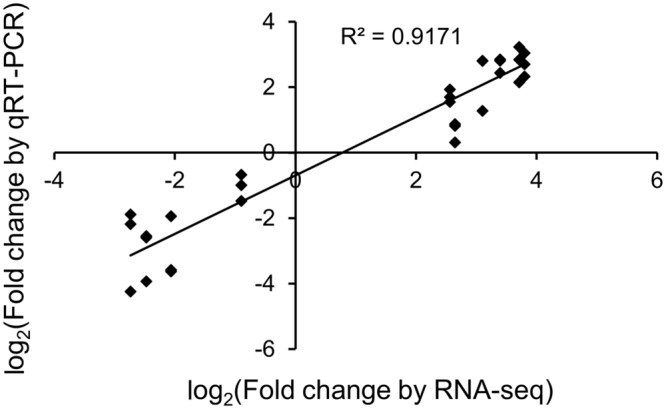
**Validation of RNA-seq differential expression results.** The correlation of JA-induced expression changes obtained from RNA-seq and qRT-PCR is shown for 10 transcripts, eight of which were randomly selected. Results for qRT-PCR are from three independent biological replicates for each transcript.

To validate RNA-seq results, the expression of eight randomly selected transcripts and two selected transcripts (discussed below) from our defined subset was assessed by qRT-PCR from control and JA-treated plants (**Figure 3**). An *R*^2^ correlation value of 0.917 was obtained, indicating high correspondence between the two methods of transcript quantitation.

### Identification and Analysis of PAP Isoforms

Following transcriptome annotation, we were able to identify assembled PAP isoforms on the basis of homology with published sequences. Six transcripts were annotated as RIPs through BLASTx and contained predicted RIP domains within their translated ORFs; their annotation information is summarized in **Table 5**. Two isoforms, PAP-I and PAP-II, had perfectly assembled ORFs. Partial transcripts of PAP-alpha and PAP-S were also identified, with 73 and 39% coverage and nearly 100% sequence identity with their respective hits. Interestingly, transcript c18776_g1_i1 had only 40% identity with PAP-alpha, its top BLASTx hit, and 86% coverage. Furthermore, the *E*-value of its identified RIP domain was more significant than that of PAP-II, which was correctly assembled. We cloned and sequenced the predicted ORF of c18776_g1_i1 from pokeweed total RNA, confirming expression of the transcript in the plant. Taken together, we hypothesize that transcript c18776_g1_i1 represents a novel PAP isoform. Transcript c115037_g1_i1, with 81% identity to PAP-I, may be another novel isoform; however, with only 38% coverage, we could not rule out the possibility that this transcript was an assembly artifact.

**Table 5:** Identification of PAP isoforms in the pokeweed mRNA transcriptome from BLASTx and Pfam annotations.

Transcript		BLASTx alignment						RIP protein domain (PF00161.14)	
ID	Length (bp)	Top hit	Gene name	Region in Query (Q) and Hit (H)	E-value	% Identity	% Coverage	Region	E-value
c3137_g1_i1	1259	RIP1_PHYAM	Antiviral protein I	Q:129–1067, H:1–313	0	100.00	100.00	28–239	3.50E–67
c3192_g1_i1	1401	RIP2_PHYAM	Antiviral protein 2	Q:173–1102, H:1–310	0	98.71	100.00	29–240	2.70E–58
**c18776_g1_i1**	**1479**	**RIPA_PHYAM**	**Antiviral protein alpha**	**Q:285–1019, H:26–277**	**2E**–**39**	**39.84**	**85.71**	**39–242**	**2.50E**–**59**
c30332_g1_i2	643	RIPA_PHYAM	Antiviral protein alpha	Q:2–643, H:62–275	5E–156	99.53	72.79	1–180	1.90E–53
c16017_g1_i1	305	RIPS_PHYAM	Antiviral protein S	Q:2–304, H:85–185	8E–67	99.01	38.70	1–101	6.10E–24
**c115037_g1_i1**	**552**	**RIP1_PHYAM**	**Antiviral protein I**	**Q:2–358, H:49–167**	**9E**–**79**	**80.67**	**38.02**	**2–106**	**3.90E**–**20**

*Putative novel PAP isoforms are indicated in bold.*

Differential expression results of the six identified PAP isoforms are provided in **Table 6**. With the exception of c18776_g1_i1, the hypothesized novel isoform, all other transcripts showed a significant increase in abundance upon JA treatment (FDR < 0.05). PAP-S had the most significant result, with a log_2_ FC of 12.07, a remarkable 4,300-fold increase. Transcript c115037_g1_i1, the other putative novel isoform, showed the highest log_2_ FC of 13.17. The expression patterns of c18776_g1_i1 and c115037_g1_i1 are distinct from PAP-alpha and PAP-I, their respective top hits, further supporting the hypothesis that they are novel isoforms. We validated the expression of c18776_g1_i1 by qRT-PCR. Although its reduction with JA treatment was not significant by RNA-seq (FDR < 0.05), qRT-PCR indicated a log_2_ FC of –1.04 (0.49 fold), which was significant (Student’s *t*-test, *p* < 0.05; **Figure 3**). The fully assembled PAP-I and PAP-II isoforms showed the lowest log_2_ FC of 3.83 and 3.11, respectively; interestingly, they were also the most abundant. Taken together, these results indicate that PAP isoforms respond differently to JA treatment, in terms of FC and abundance.

**Table 6:** Differential expression of PAP isoforms.

Transcript ID	Top BLASTx Hit	log_2_FC	log_2_FPKM E	log_2_FPKM JA	FDR
c16017_g1_i1	RIPS_PHYAM	12.07	-1.79	10.55	1.87E–96
**c115037_g1_i1**	**RIP1_PHYAM**	13.17	-3.32	7.16	**1.37E**–**75**
c30332_g1_i2	RIPA_PHYAM	7.77	-2.20	5.62	2.12E–42
c3137_g1_i1	RIP1_PHYAM	3.83	10.41	14.35	1.76E–39
c3192_g1_i1	RIP2_PHYAM	3.11	9.97	13.18	3.95E–26
**c18776_g1_i1**	**RIPA_PHYAM**	-0.90	7.69	6.88	**6.81E**–**02**

*Transcripts are listed in order of decreasing significance. Putative novel isoforms are indicated in bold.*

### Discovery of Natural Antisense Transcripts in Pokeweed

Owing to strand-specific sequencing of the pokeweed mRNA transcriptome, we were able to identify putative NATs. This involved performing a BLASTn search of the assembled transcriptome against its reverse complement to find transcript pairs having significant sequence complementarity (*E* < 0.001). In total, 52,305 NAT pairs were detected, although this number is an under-representation because only the best-matching partner for each transcript was retained in our analysis.

**FIGURE 4:**
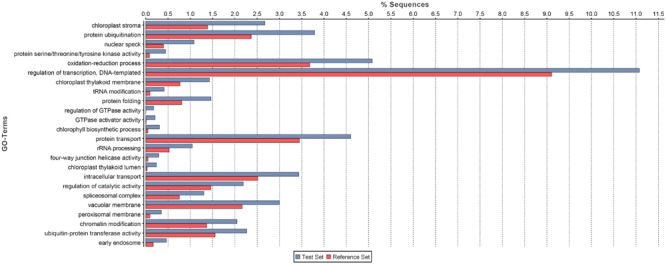
**Functional analysis of NATs in pokeweed.** GO enrichment analysis was conducted on all NATs (Test Set) against the raw pokeweed transcriptome assembly (Reference Set). Enriched terms (FDR < 0.001) are listed in order of increasing significance (bottom to top).

We conducted GO enrichment analysis to investigate the potential functional roles of NATs in pokeweed. As shown in **Figure 4**, NATs were enriched in 24 GO terms relative to all pokeweed transcripts from the raw assembly (FDR < 0.001). Of these, “chloroplast stroma” was most significant and three other chloroplast-related terms were also enriched: “chloroplast thylakoid membrane,” “chlorophyll biosynthetic process,” and “chloroplast thylakoid lumen.” From the 52,305 pairs of NATs, 2,502 transcripts were differentially expressed (FDR < 0.001, FC ≥ 4). Of the differentially expressed NATs, 88 pairs involved both partners, suggesting co-regulation in response to JA. The 88 pairs were enriched in three GO terms relative to all pokeweed transcripts: “nitrate assimilation,” “nitrate reductase (NADH) activity,” and “molybdopterin cofactor binding” (FDR < 0.001). The transcripts relating to these terms were annotated as NRT1/PTR FAMILY members and nitrate reductases. Therefore, it appears that in pokeweed, NATs in general are important in the chloroplast, while JA-responsive NATs may regulate nitrate metabolism.

**FIGURE 5:**
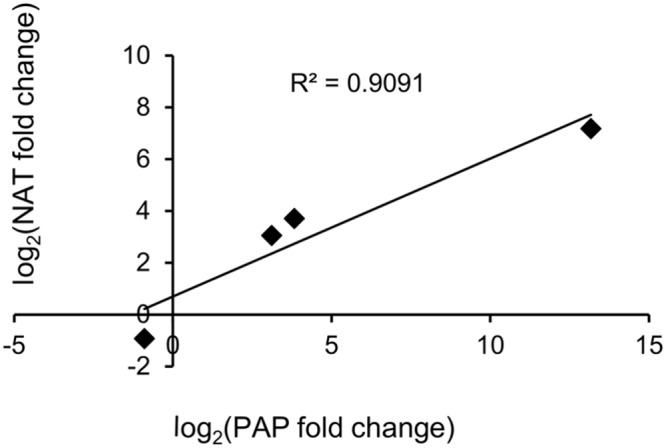
**Expression of PAP sense and antisense transcripts.** The correlation of JA-induced expression changes obtained by RNA-seq is shown for each PAP isoform and its corresponding NAT.

As shown in **Table 7**, four PAP isoforms had significant NAT counterparts (PAP-I, PAP-II, and the two potentially novel isoforms). Each NAT showed perfect alignment identity and nearly full-length coverage with its respective PAP isoform. Interestingly, the expression of PAP isoforms and their corresponding NATs showed a strong positive correlation, with an *R*^2^ value of 0.909 (**Figure 5**). We cloned and sequenced the PAP-I NAT (c61645_g1_i1) from pokeweed total RNA to validate its expression. Furthermore, qRT-PCR of PAP-I NAT from JA-treated plants indicated a significant increase of 6.68 fold (log_2_FC of 2.74; Student’s *t*-test, *p* < 0.05; **Figure 3**). These results confirm the expression of a JA-responsive transcript that is antisense to the PAP-I sequence. In addition, they suggest that certain PAP isoforms may be regulated by NATs, which would constitute a novel form of PAP gene regulation.

**Table 7:** Identification and expression of putative PAP natural antisense transcripts.

PAP isoform transcript	NAT	NAT length (bp)	% ID	% Coverage	Aligned regions of PAP (P) and NAT (N)	log_2_FC	log_2_FPKM E	log_2_FPKM JA	FDR
c3137_g1_i1	c61645_g1_i1	1260	100	100.08	P:22–1259, N:1–1238	3.71	5.08	8.90	1.95E – 27
c3192_g1_i1	c37772_g1_i1	1180	99.92	84.23	P:85–1264, N:1–1180	3.05	3.64	6.80	1.16E–17
c18776_g1_i1	c24011_g1_i1	1319	100	89.18	P:43–1361, N:1–1319	–0.92	1.76	0.93	0.35936
c115037_g1_i1	c21462_g1_i2	551	100	99.82	P:2–552, N:1–551	7.18	–3.32	1.15	2.44E–06

## Discussion

Through transcriptome assembly, annotation and differential expression analysis, we identified genes that are significantly affected by JA and could mediate defense against pathogens and herbivores in *P. americana*. We validated the existence of several previously reported PAP isoforms and characterized their differential expression patterns. This work also led to the discovery of a potentially novel PAP isoform and identification of NATs that may regulate PAP gene expression.

### Construction of a High-Quality Pokeweed Reference Transcriptome

We generated a robust pokeweed mRNA transcriptome by combining strand-specific, paired-end sequencing reads from biological replicates of JA-treated and control plants. Over 400 million processed reads were leveraged for assembly, which is considered very deep sequencing (Haas et al., 2013). Comparison of our assembly with sugar beet, a well-studied plant from the same taxonomic order as pokeweed, reveals important similarities to help validate our results. For example, *de novo* assembly of the sugar beet mRNA transcriptome yielded a total of 225,385 transcripts from 165,742 unique loci, providing an N50 value of 1,185 bp (Mutasa-Göttgens et al., 2012). These statistics are comparable to our pokeweed *Raw* assembly, which had 216,891 transcripts, 177,709 unigenes and an N50 value of 1,168 bp. Furthermore, others reported that approximately 80% of sugar beet unigenes are between 200 and 500 bp in length (Fugate et al., 2014), in agreement with the abundance of short transcripts we detected in pokeweed. The enrichment of short transcripts seems to be a general trend amongst *de novo* assembled transcriptomes, perhaps due to the assembly algorithm which relies on short overlapping sub-sequences known as k-mers (Ranjan et al., 2014). Through BLASTx searches against public sequence databases, we were able to annotate 59,096 transcripts. This is in line with other studies of non-model plants, with examples of approximately 40–50,000 annotated transcripts or unigenes (Farrell et al., 2014; Jin et al., 2014; Ranjan et al., 2014). Taken together, reports from other plants support the validity of our pokeweed assembly.

### Discovery of Putative Defense Genes in Pokeweed

We were interested in identifying JA-responsive genes in pokeweed because this hormone mediates broad-spectrum defense. We reported 165 annotated transcripts specific to JA treatment. Many of these transcripts encoded factors involved in JA biosynthesis, JA signaling and JA-mediated defense, which have been identified in other transcriptome-wide studies involving transgenic plants deficient in JA biosynthesis or signaling (Abuqamar et al., 2006; Ku et al., 2011; Yan et al., 2014). Identification of these JA responses allows us to integrate PAP within broader defense pathways of pokeweed.

We also sought to identify genes other than PAP that may contribute to defense in this plant. One interesting candidate was *intracellular ribonuclease LX*, which showed a 14,664-fold increase with JA. Such changes are not uncommon in plants responding to biotic and abiotic stresses (Liu et al., 2013; Pierce and Rey, 2013; Li et al., 2014). This gene, characterized in tomato, is involved in programmed cell death responses including senescence and is thought to contribute to nutrient recycling by mediating RNA turnover (Lehmann et al., 2001; Lers et al., 2006). It is well-established that jasmonate is associated with localized cell death as part of the hypersensitive response in plants, providing a possible explanation for the increased expression of this gene in JA-treated plants. Future work will investigate if this enzyme has a role in pokeweed defense, as pathogen-responsive RNases have been reported in other plants (Galiana et al., 1997; Ayashi et al., 2003).

Another markedly up-regulated gene in our study was annotated as *bark storage protein A* and is involved in nitrogen storage in senescing leaves (Wetzel et al., 1989; Coleman et al., 1991). Storage proteins have previously been linked to jasmonate-mediated responses, such as VSP2 in *Arabidopsis*, an anti-insect acid phosphatase (Liu et al., 2005). A final candidate is *defensin-like protein*, which, in addition to being differentially expressed, was also the most abundant transcript in JA-treated plants. In *Arabidopsis*, the defensin PDF1.2 is a well-established marker of the ethylene and jasmonate signaling pathway that mediates resistance to pathogens (Manners et al., 1998; Penninckx et al., 1998). Identification of JA-responsive genes in pokeweed will allow a broader characterization of factors involved in defense in this plant. Furthermore, since only 38% of expressed pokeweed transcripts were annotated, this non-model plant transcriptome represents a source of new genes that could have agricultural benefits.

### Characterization of PAP Isoforms and their Differential Expression Patterns

Through transcriptome annotation, we confirmed the presence of the following isoforms in pokeweed leaf tissue from 4-leaf plants: PAP-I, PAP-II, PAP-S, and PAP-alpha. Based on previous reports, PAP-I is expressed in spring leaves (Irvin, 1975), PAP-II in early summer leaves (Irvin et al., 1980), PAP-S in seeds (Barbieri et al., 1982) and PAP-alpha in various tissues (Kataoka et al., 1992). We also presented sequence and qRT-PCR evidence to support the existence of a novel PAP isoform. Our finding that PAP-I was the most abundant isoform in young plants, followed by PAP-II, agrees with their documented temporal profiles. Furthermore, the high abundance of the isoforms corresponds with the report that PAP comprises up to 0.5% of total soluble protein in leaves (Bonness et al., 1994). Interestingly, we only found one annotated PAP-S isoform. This disagrees with a previous finding that PAP-S is a mix of two seed isoforms, PAP-S1 and PAP-S2, having 85% nucleotide sequence identity (Honjo et al., 2002). Our result was not likely due to the assembly algorithm, since Trinity is able to differentiate between sequences that are up to 95% identical (Grabherr et al., 2011). This does not preclude the possibility that two PAP-S isoforms exist in seeds, as we only investigated leaf tissue. Furthermore, we did not identify PAP-R or PAP-III isoforms, which have been purified from root tissue and late summer leaves, respectively (Bolognesi et al., 1990; Rajamohan et al., 1999).

RNA-seq analysis allowed us to compare the expression patterns of multiple PAP isoforms simultaneously. This level of distinction in a single experiment has not yet been possible, likely due to difficulties related to high sequence identity amongst isoforms. With the exception of PAP-II, the nucleotide sequences of isoforms are >70% identical. In the current study, we found that all isoforms other than c18776_g1_i1 were significantly up-regulated with JA. PAP isoforms exhibited differences in terms of abundance and FC. Such differences could indicate that they have specialized roles in defense, or more generally within the plant. For example, analysis of two RIP isoforms in spinach revealed that one was highly expressed in embryos and not responsive to salicylic acid, while the other showed weak expression throughout the plant but was induced with salicylic acid (Kawade and Masuda, 2009). Authors suggested that the two RIPs may have different functions, one in embryogenesis and the other in defense. In pokeweed, PAP isoforms differ in their ability to depurinate eukaryotic and prokaryotic ribosomes *in vitro* (Honjo et al., 2002). A comprehensive RNA-seq study that includes different developmental stages and stresses would allow further insight into the possible roles of PAP isoforms.

### Natural Antisense Transcripts in Pokeweed

Natural antisense transcripts are pairs of endogenous transcripts with high sequence complementarity, capable of forming double-stranded RNA and affecting gene expression in *cis* or *trans*. *Cis*-NATs are transcribed from the same genomic locus but opposite orientations and have perfect sequence complementarity, while *trans*-NATs are derived from different loci. Our sequence analysis identified over 52,000 NAT pairs in pokeweed. A recent study identified 37,238 NAT pairs in *Arabidopsis* and an astonishing 70% of annotated mRNAs were associated with antisense transcripts (Wang et al., 2014). Furthermore, 60% of NAT pairs were comprised of fully overlapping transcripts. In pokeweed, we found perfectly identical, fully overlapping antisense transcripts of PAP-I, PAP-II, c18776_g1_i1, and c115037_g1_i1. The fact that each NAT was perfectly complementary to its respective isoform suggests that they act in *cis*. We also found that PAP NATs and their corresponding sense transcripts had positively correlated responses to JA, indicating that NATs may regulate PAP expression. This agrees with studies in humans and other mammals which report significant positive correlations between sense and antisense transcription (Oeder et al., 2007; Ling et al., 2013). Fewer genome-wide studies of NAT transcription have been performed for plants. In *Arabidopsis*, a study of light-responsive long non-coding NATs identified 626 positively correlated and 766 negatively correlated pairs (Wang et al., 2014). NATs have been proposed to modulate gene expression at various levels, including transcription, post-transcriptional small RNA interference, mRNA splicing and RNA stability (Pelechano and Steinmetz, 2013; Zhang et al., 2013; Liu et al., 2015). Future work will investigate mechanisms by which NATs may regulate PAP expression.

Apart from PAP regulation, we conducted preliminary investigations into the broader roles of NATs in pokeweed. As a group, NATs were enriched in several GO terms relating to the chloroplast. Although the implications of this are unknown, it agrees with a previous report from *Arabidopsis* that the chloroplast encodes a diverse group of antisense RNAs mapping to protein-coding genes (Hotto et al., 2011). Approximately 2,500 NATs were JA-responsive and 88 pairs were considered biologically relevant because both sense and antisense strands were differentially expressed. These NAT pairs were enriched in GO terms relating to nitrate metabolism and the involved transcripts were annotated as NRT1/PTR FAMILY members and nitrate reductases. These enzymes have well-established roles in nitrate assimilation, which involves the reduction of nitrate to nitrite, followed by ammonium, which is ultimately incorporated into amino acids for plant growth (Dechorgnat et al., 2010). Importantly, the reduction of nitrite to ammonium occurs in chloroplasts, further supporting the connection between NATs and this specific organelle. Furthermore, NRT1/PTR FAMILY members capable of transporting jasmonoyl-isoleucine, the bioactive form of jasmonate, were recently identified (Chiba and Shimizu, 2015). Given that plant stress decouples nitrate assimilation and photosynthesis, mediated by the jasmonate/ethylene signaling pathway (Zhang et al., 2014), NATs in pokeweed may be involved in regulating the trade-off between stress response and plant growth.

## Summary and Relevance

Here, we have assembled and annotated the pokeweed leaf mRNA transcriptome under JA treatment. In addition to the identification of many differentially expressed transcripts, we also characterized the expression of multiple PAP isoforms through RNA-seq analysis, including a potentially novel isoform of PAP. Finally, we present the first report of NATs in the pokeweed plant and confirmed expression of a PAP-NAT, which may indicate a novel form of RIP gene regulation. The pokeweed transcriptome will enable further investigations into the robust defense strategies of this species and other Phytolaccaceae family members. Heterologous expression of PAP has already been applied successfully to produce virus resistant plants (Lodge et al., 1993; Zoubenko et al., 1997; Wang et al., 1998; Dai et al., 2003). Pokeweed accumulates high levels of metal from contaminated soils but little is known about the genes mediating this tolerance (Dou et al., 2009; Zhao et al., 2011, 2012). With a complete repository of pokeweed mRNA sequences, we anticipate the discovery of beneficial genes in this plant that could improve the resiliency of agricultural crops.

## Author Contributions

KN and KH conceived the design and coordination of the study. KN performed bioinformatic analyses, statistical testing and drafted the manuscript. AK carried out qRT-PCR validations and cloning. KH edited the manuscript. All authors read and approved the final manuscript.

## Conflict of Interest Statement

The authors declare that the research was conducted in the absence of any commercial or financial relationships that could be construed as a potential conflict of interest.

## Abbreviations

E: ethanol
FC: fold change
FDR: false discovery rate
FPKM: fragments per kilobase per transcript per million mapped reads
GO: gene ontology
JA: jasmonic acid
NAT: natural antisense transcript
ORF: open reading frame
PAP: pokeweed antiviral protein
RIP: ribosome inactivating protein
TMM: trimmed mean of *m*-values
