# Supplementary figures and images for: The Pokeweed Leaf mRNA Transcriptome and Its Regulation by Jasmonic Acid
Source: Front Plant Sci. 2016 Mar 16;7:283. doi: 10.3389/fpls.2016.00283 (PMC4792876; doi:10.3389/fpls.2016.00283)

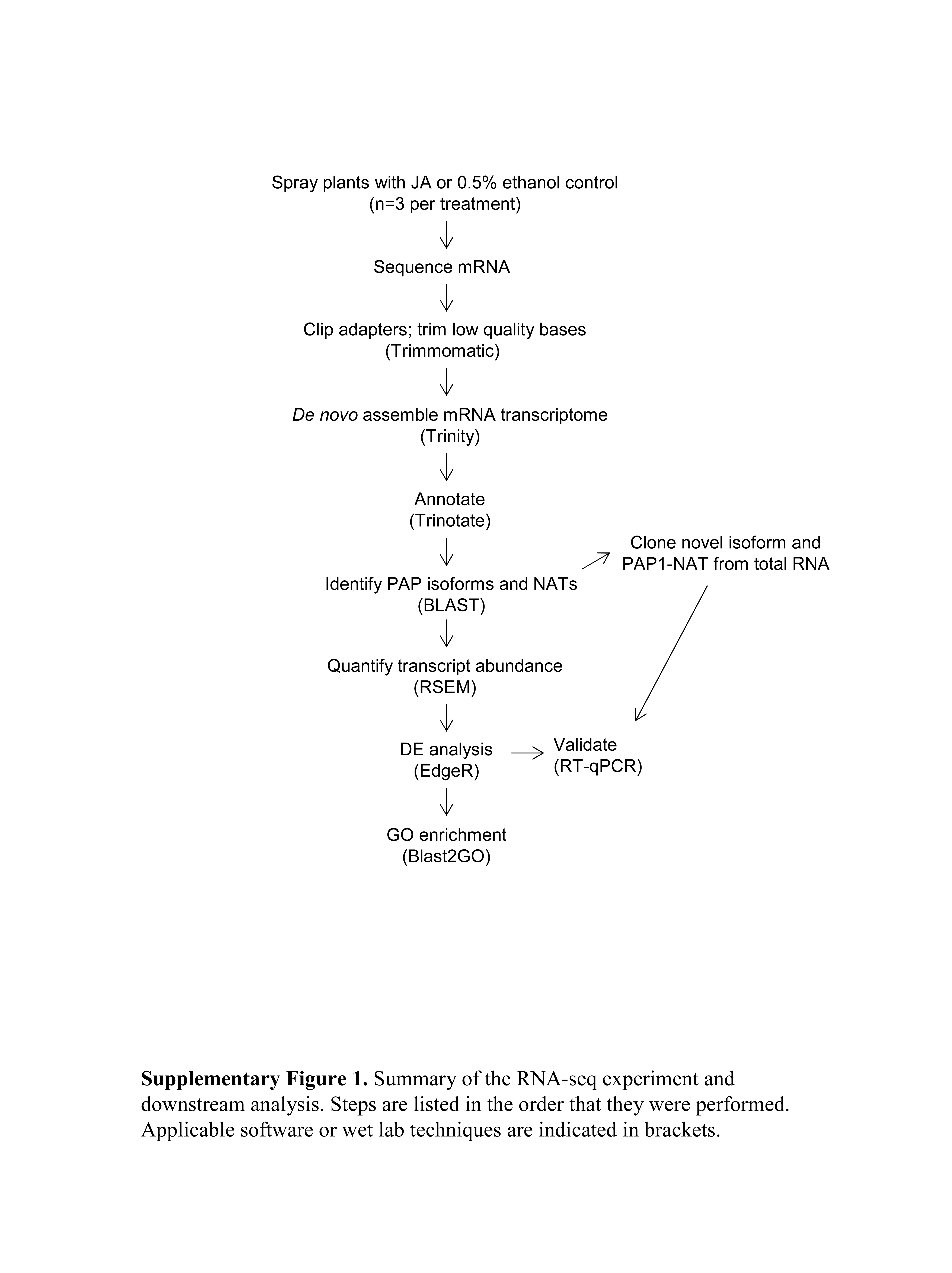

Supplement: Supplementary file 7 [file Image_1.TIF]

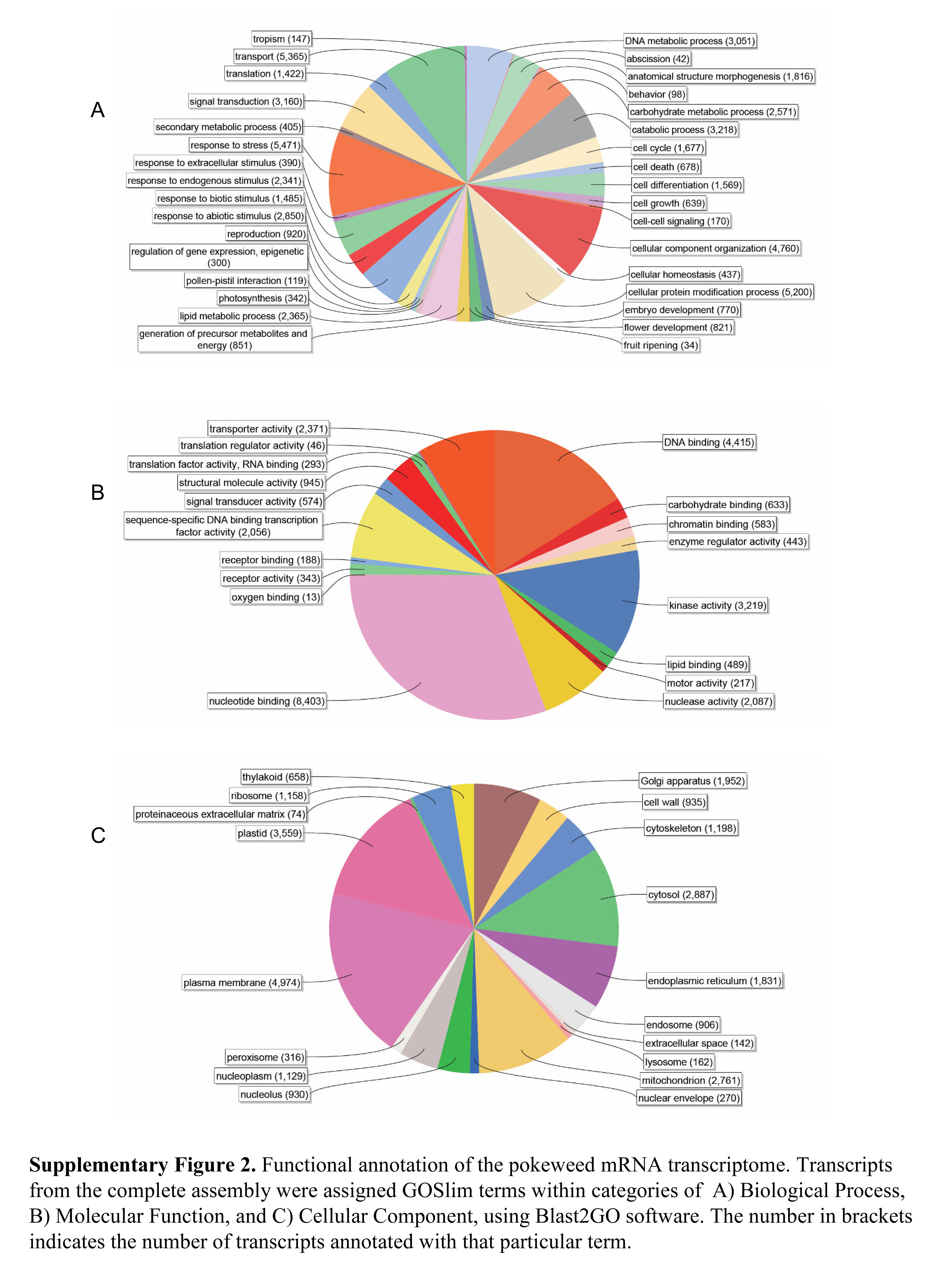

Supplement: Supplementary file 8 [file Image_2.TIF]

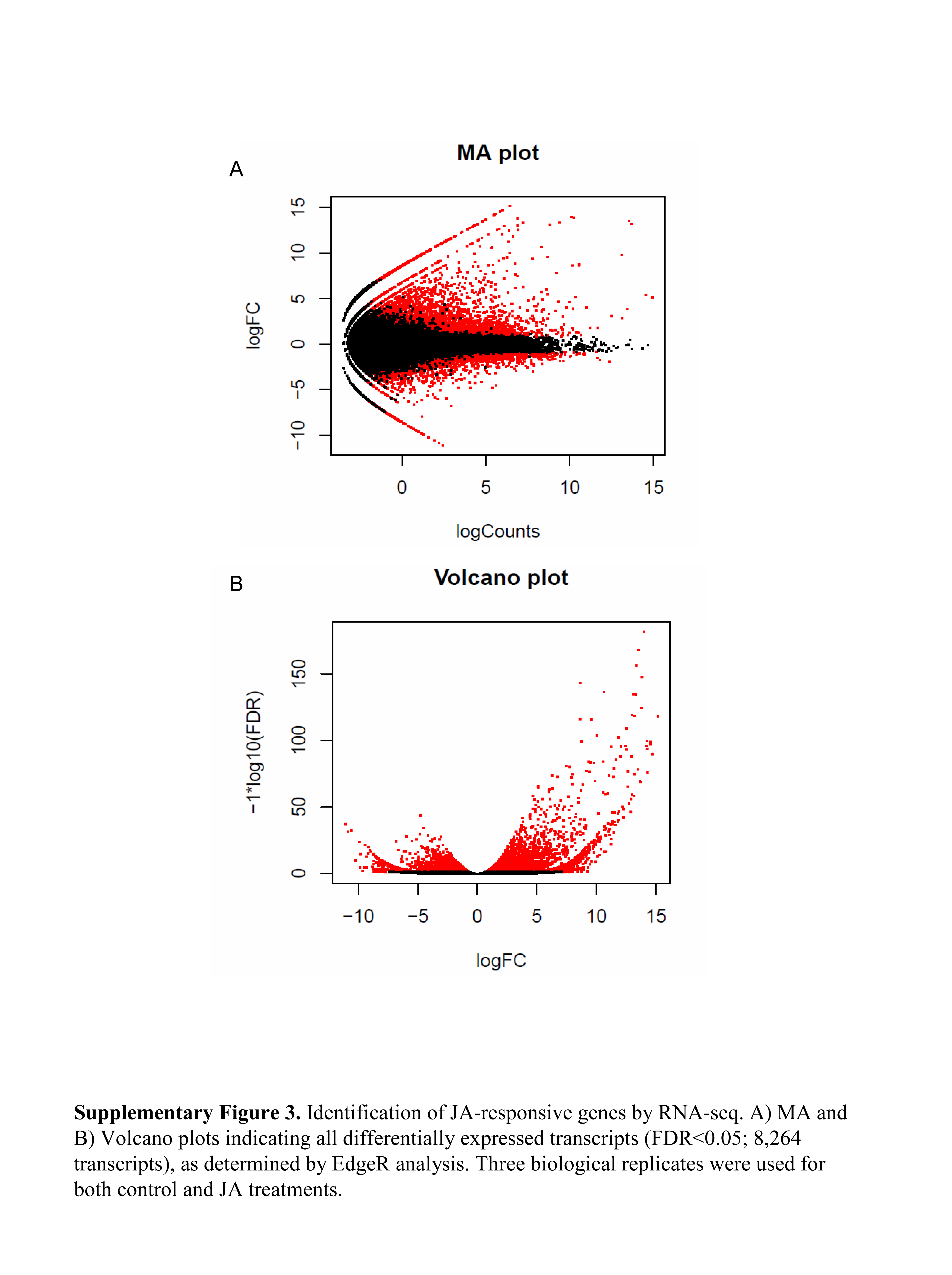

Supplement: Supplementary file 9 [file Image_3.TIF]

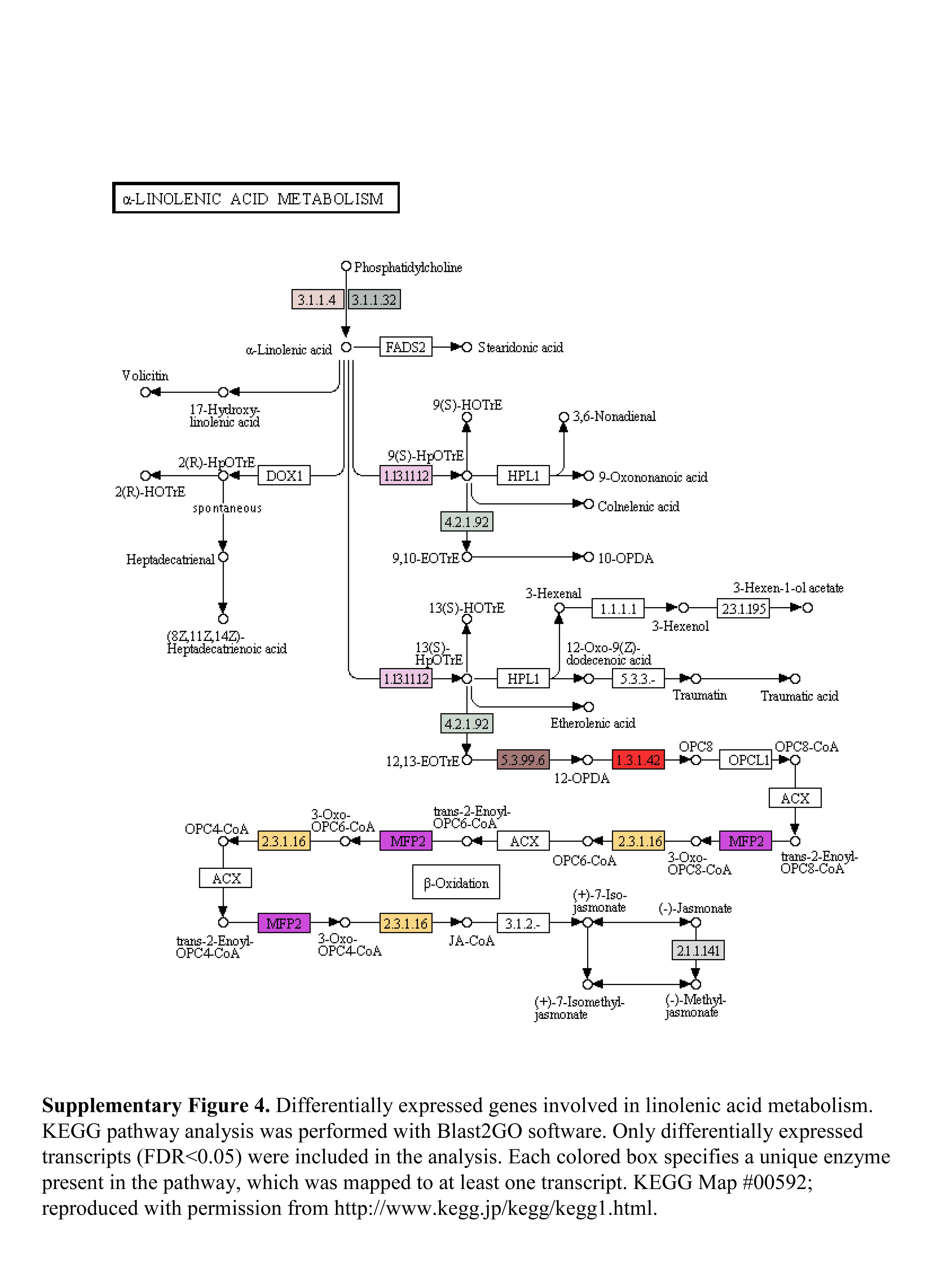

Supplement: Supplementary file 10 [file Image_4.TIF]
